# Lattice Model Results for Pattern Formation in a Mixture with Competing Interactions

**DOI:** 10.3390/molecules29071512

**Published:** 2024-03-28

**Authors:** Andres De Virgiliis, Ariel Meyra, Alina Ciach

**Affiliations:** 1 Instituto de Física de Líquidos y Sistemas Biológicos, Facultad de Ciencias Exactas-UNLP-CONICET, La Plata 1900, Argentina; adevir@iflysib.unlp.edu.ar (A.D.V.); agmeyra@gmail.com (A.M.); 2 Departamento de Ciencias Básicas, Facultad de Ingeniería, Universidad Nacional de La Plata, La Plata 1900, Argentina; 3 Departamento de Ingeniería Mecánica, Facultad Regional La Plata, Universidad Tecnológica Nacional, La Plata 1900, Argentina; 4 Institute of Physical Chemistry, Polish Academy of Sciences, 01-224 Warsaw, Poland

**Keywords:** colloidal self-assembly, spontaneous pattern formation, inhomogeneous mixtures, Monte Carlo simulations, self-assembled stripes, theory for mixtures with competing interactions

## Abstract

A monolayer consisting of two types of particles, with energetically favored alternating stripes of the two components, is studied by Monte Carlo simulations and within a mesoscopic theory. We consider a triangular lattice model and assume short-range attraction and long-range repulsion between particles of the same kind, as well as short-range repulsion and long-range attraction for the cross-interaction. The structural evolution of the model upon increasing temperature is studied for equal chemical potentials of the two species. We determine the structure factor, the chemical potential–density isotherms, the specific heat, and the compressibility, and show how these thermodynamic functions are associated with the spontaneous formation of stripes with varying degrees of order.

## 1. Introduction

Self-assembly on the length scale of nanometers or micrometers is quite common in biological and soft matter systems [1,2,3,4,5,6,7,8,9,10,11,12,13,14,15,16,17,18]. Various spontaneously formed patterns made by proteins, nanoparticles, core–shell particles, or quantum dots appear on biological membranes, in Langmuir monolayers or at solid substrates [19,20,21,22,23,24,25,26]. Patterns spontaneously occurring in nature can, in principle, be self-assembled in the laboratory on a desired length scale, as periodically distributed elements can range from atoms to colloid particles or clusters of such particles when the interactions and thermodynamic conditions are properly tuned. The aim of such studies is the production of metamaterials with various optical, electromagnetic, acoustic, and thermal properties. Among the possible patterns, stripes or layers with different thicknesses often appear in one-component systems of particles [5,7,25], or in mixtures [20,27,28,29,30]. Ordered thin stripes of alternating components can find various technological applications, such as in photonic crystals [31,32] or optoelectronic devices [33]. Fundamental knowledge concerning conditions that support the spontaneous formation of ordered stripes is important. The thermodynamically stable state results from the competition between the minimization of energy, leading to an ordered structure, and the maximization of entropy, leading to a disordered one. The balance between these two tendencies is crucial because it determines the temperature at which patterns with a minimal number of defects are formed. This interplay between the energy and the entropy in systems with spontaneous pattern formation leads to more complex phenomena than in simple fluids, and phases with different symmetries and different degrees of order on the microscopic, mesoscopic, and macroscopic length scales can occur.

Due to the inherent complexity of the interactions involved, which makes it difficult to exert precise control over the relevant parameters, the main features of the fundamental processes described above can hardly be addressed in laboratory experiments. On the other hand, basic aspects of the structural changes in the self-assembling systems can be studied by considering simplified models for fluids [34]. Thus, in this work, we study a simple triangular lattice model for a binary mixture with competing interactions in the case of equal chemical potentials of the two components.

Within this context, self-assembled patterns made of alternating stripes can be induced by different types of competing interactions between the constitutive particles. In the simplest model introduced in Ref. [35] and studied recently in Ref. [27], the only interaction added to hard spheres was the square-well attraction between different components. In another model inspired by oppositely charged hydrophilic and hydrophobic particles in a solvent close to the miscibility critical point, such particles interact via the short-range attraction and long-range repulsion (SALR) potential, V(r), and the cross interaction is −V(r), i.e., the potential between different particles is repulsive at short distances and attractive at long distances. The above model is termed ‘two mermaids and a peacock’ because of the attractive head and repulsive tail in V(r), and the repulsive head and attractive tail in −V(r). It is worth mentioning that competing interactions of the above form can be present between charged transmembrane proteins, where the short-range interaction is induced by concentration fluctuations in the multicomponent lipid bilayer in living organisms [36].

In the one-component SALR (mermaid) systems, the sequence of ordered phases is universal [34], and only the size of the aggregates depends on the strengths and ranges of the attractive and repulsive parts of the potential. In contrast, in one-component systems with repulsion at short distances and attraction at large distances (peacock), the patterns can be quite different for different forms of short-range repulsion [22,25,37]. So, it is not clear how sensitive the pattern formation in the ‘two mermaids and a peacock’ model is to the detailed shapes of the interactions.

So far, a few variants of the ‘two mermaids and a peacock’ model have been introduced [28,30,38]. It was discovered that in continuous models where the repulsive part of V(r) is weak but long-ranged, the disordered phase coexists with alternating stripes or layers in 2D and 3D systems for equal chemical potentials of the two components. Only the thickness of the layers can be different. In a triangular lattice model where the repulsive interaction between like particles is strong but short-ranged, stripes have been found for large chemical potentials. The above stripe (or lamellar) phase coexists at moderate chemical potentials with parallel chains of alternating clusters of the two components separated by empty layers, and thin one-component zig-zag chains separated from chains of the other component by voids. These two phases coexist in turn with the disordered gas phase at still smaller chemical potentials. The above structures, however, were found in a study limited to very low temperatures [38].

At zero temperature, the equilibrium phases are perfectly ordered. However, when the temperature is quenched to very small values at large chemical potentials, mesoscopic domains with stripes of different orientations are formed. At very low *T*, the energetic barrier for the reorientation of stripes in neighboring domains is large, making it practically impossible to reach the equilibrium state within a reasonable time frame. As a result, perfectly ordered mesoscopic domains with randomly distributed orientations of the stripes in these domains are formed [38]. Thus, on a larger length scale, the system is isotropic. Another problem is that at room temperature, the interactions between particles must be very strong to obtain the very small ratio of the thermal energy and the potential energy in the optimal configuration.

Increasing temperature can, on one hand, lead to local defects in the structure, such as fractures, branching, and dislocations of the stripes. On the other hand, with higher *T*, the reorientation of the stripes is more probable and the stripes may assume the same orientation throughout the whole sample. Thus, more global order can be achieved at the cost of the formation of local defects. The above speculations, however, need to be verified, and a precise analysis of structural evolution is necessary. We should note that, in general, in self-assembling systems, heating for fixed chemical potential or density can lead to transitions between phases with different symmetries and degrees of order before the transition to the disordered phase takes place. Thus, more complex phenomena than in simple fluids can be expected for the self-assembling mixture on a pre-patterned surface. For the above reasons, it is important to investigate the disordering effect of temperature on the ordered patterns determined in Ref. [38], and this is the purpose of our study.

In Section 2, we introduce and discuss the model, and present the theoretical and simulation methods. We limit ourselves to a symmetrical case of equal chemical potentials of the two components. Section 3.1 shows the theoretical results for the boundary of stability of the disordered phase in the mean field (MF) approximation and the correlation function in the self-consistent theory accounting for the variance in the local concentration. In Section 3.2, we first briefly summarize the results of Ref. [38] for very low temperatures, and then present our simulation results for the structure factor, density, specific heat, and compressibility for a broad temperature range. The discussion and conclusions are presented in Section 4 and Section 5.

## 2. Model and Methods

### 2.1. The Model

We assume that particles occupy cells of a triangular lattice, and multiple occupancies of the lattice cells are forbidden. The lattice constant, *a*, is identified with the size of the hard cores of the particles, and we choose *a* for the length unit. The lattice sites are denoted by $x=x_{1}{\hat{e}}_{1}+x_{2}{\hat{e}}_{2}$, with ${\hat{e}}_{1}$, ${\hat{e}}_{2}$ denoting the unit lattice vectors on the triangular lattice with the scalar product in the Euclidean plane, ${\hat{e}}_{1}\cdot {\hat{e}}_{2}=\sqrt{3}/2$. The third lattice vector is ${\hat{e}}_{3}={\hat{e}}_{2}-{\hat{e}}_{1}$. For distances r>1 in *a*-units, the interaction potential between the particles separated by the distance, Δx, is as follows:

(1) $$V_{ij}\left(\Delta x\right)=\left\{\begin{array}{c} V(\Delta x)\;\mathrm{for}i=j\;\\ -V(\Delta x)\;\mathrm{for}i\neq j\; \end{array}\right.$$

where i,j=1,2 refer to the first and second components, and

(2) $$V\left(\Delta x\right)=\left\{\begin{array}{c} -J_{1}\;\mathrm{for}|\Delta x|=1,\;(\mathrm{for}\mathrm{nearest}\mathrm{neighbors})\\ +J_{2}\;\mathrm{for}|\Delta x|=2,\;(\mathrm{for}\mathrm{third}\mathrm{neighbors})\\ 0\;\mathrm{otherwise} \end{array}\right.$$

**Figure 1:**
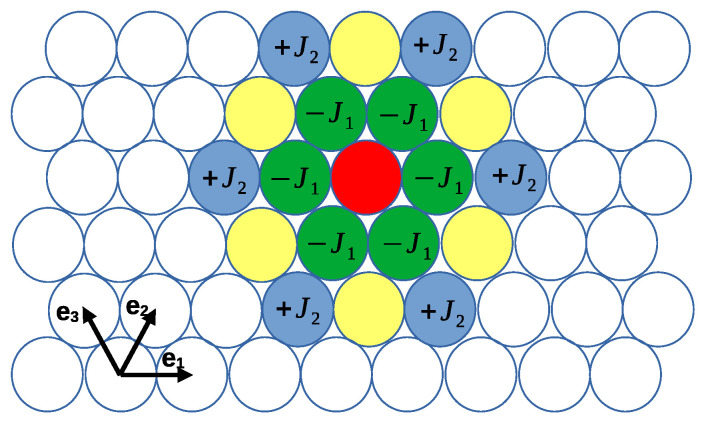
Cartoon showing the lattice vectors ${\hat{e}}_{i}$ and energy of the interactions between a particle occupying the red cell with particles of the same species occupying neighboring cells, as indicated (see Equation (2)). The interactions of the particle in the red cell with particles of the other component have opposite signs. The interaction changes sign for the distance $|{\hat{e}}_{1}+{\hat{e}}_{2}|$ (yellow cells), and the interaction with the particles occupying the remaining cells is negligible.

$-J_{1}$ and $J_{2}$ represent the energy of the attraction and repulsion between like particles for |Δx|=1 and |Δx|=2, respectively. We use the dimensionless energy, $\bar{V}\left(\Delta x\right)=V\left(\Delta x\right)/J_{1}$, and dimensionless temperature, $\bar{T}=1/\bar{\beta }=k_{B}T/J_{1}$, where $k_{B}$ is the Boltzmann constant, along with dimensionless chemical potentials, ${\bar{\mu }}_{i}=\mu_{i}/J_{1}$. The relevant dimensionless parameter associated with the shape of the interactions is the ratio $J=J_{2}/J_{1}$ between the third and the nearest neighbor potentials (see Figure 1).

We will consider open systems that are in contact with a reservoir of particles so that at T=0, the equilibrium state corresponds to the minimum of the thermodynamic Hamiltonian, which has the following form:

(3) $$H\left[{\hat{\rho }}_{1},{\hat{\rho }}_{2}\right]=\frac{1}{2}\sum_{x}\sum_{x^{\prime }}{\hat{\rho }}_{i}\left(x\right)V_{ij}\left(x-x^{\prime }\right){\hat{\rho }}_{j}\left(x^{\prime }\right)-\mu_{i}\sum_{x}{\hat{\rho }}_{i}\left(x\right)$$

where $\sum_{x}$ is made over all lattice cells, and the summation convention for repeated indexes is used. Here, ${\hat{\rho }}_{i}\left(x\right)$ is the occupation number for the *i*-th type of particles, i.e., ${\hat{\rho }}_{i}\left(x\right)=1$, or 0 if the site x is occupied or not by the *i*-th type particle, respectively. Multiple occupation of the cells is avoided by applying the condition ${\hat{\rho }}_{1}\left(x\right)\;{\hat{\rho }}_{2}\left(x\right)=0$. For T>0, the probability of particular occupation $\left\{{\hat{\rho }}_{1}\left(x\right),{\hat{\rho }}_{2}\left(x\right)\right\}$ of all lattice cells is proportional to $\exp(-\beta H\left[{\hat{\rho }}_{1},{\hat{\rho }}_{2}\right])$.

An important feature of the potential (2) is its shape in the Fourier representation,

(4) $$\begin{array}{ccc} \beta \tilde{V}\left(k\right) & = & \sum_{x}\beta V\left(x\right)e^{ik\cdot x}\\ & = & 2\bar{\beta }\left[J\left(\cos\left(2k_{1}\right)+\cos\left(2k_{2}\right)+\cos\left(2\left(k_{1}-k_{2}\right)\right)\right)-\cos\left(k_{1}\right)-\cos\left(k_{2}\right)-\cos\left(k_{1}-k_{2}\right)\right], \end{array}$$

where the vectors on the reciprocal lattice are $k=k_{1}{\hat{f}}_{1}+k_{2}{\hat{f}}_{2}$, with the scalar product on the Euclidean plane being ${\hat{e}}_{i}\cdot {\hat{f}}_{j}=\delta_{ij}^{Kr}$, where $\delta_{ij}^{Kr}$ is the Kronecker delta, and $|{\hat{f}}_{i}|=2/\sqrt{3}$. $\tilde{V}\left(k\right)$ takes the global minimum at $k_{b}^{\left(1\right)}=k_{b}{\hat{f}}_{1}+\frac{k_{b}}{2}{\hat{f}}_{2}=k_{b}{\hat{e}}_{1}$. Because of the symmetry of the lattice, $\tilde{V}\left(k\right)$ takes the minimum for the 6 vectors, as follows:

(5) $$k_{b}^{\left(j\right)}=\pm k_{b}{\hat{e}}_{j}$$

with j=1,2,3. The wavenumber $k_{b}$ is given by [6]

(6) $$\cos\left(\frac{k_{b}}{2}\right)=\frac{J+\sqrt{J^{2}+2J}}{4J},$$

and $k_{b}>0$ for J>1/4. When $\tilde{V}\left(k\right)$ takes a negative minimum for $k_{b}\neq 0$, spontaneous inhomogeneities with the wavelength determined by $k_{b}$ lead to a decrease in internal energy.

In this article, we focus on the symmetrical case of equal chemical potentials of the two components, $\mu_{1}=\mu_{2}=\mu$ and J=3, first introduced in Ref. [7], and study the effect of increasing temperature on the patterns that were found at very low temperatures.

### 2.2. The Theoretical Methods

In theoretical studies, we limit ourselves to the disordered phase, where the ensemble-averaged densities, $\langle {\hat{\rho }}_{i}\left(x\right)\rangle$, do not depend on the position x. Importantly, $\langle {\hat{\rho }}_{i}\left(x\right)\rangle =const.$ when the particles are either homogeneously distributed or form aggregates that can assemble or disassemble, and occupy different positions in different inhomogeneous microscopic configurations. We use the formalism developed for systems with spontaneous inhomogeneities in refs. [35,39,40]. In this mesoscopic theory, we consider the grand potential functional of the concentration and the total density fields defined as follows:

(7) $$c\left(x\right)=\rho_{1}\left(x\right)-\rho_{2}\left(x\right),\;\rho \left(x\right)=\rho_{1}\left(x\right)+\rho_{2}\left(x\right),$$

with $\rho_{i}\left(x\right)$ denoting the density of the *i*-th component in the cell x. The above fields become equal to the average concentration and density when they correspond to a minimum of the appropriate grand potential functional. The exact functional is not known and we consider commonly used approximations.

In the MF approximation, the grand potential functional for this model takes the following form for $\mu_{1}=\mu_{2}=\mu$:

(8) $$\beta \Omega^{MF}\left[c,\rho \right]=\frac{1}{2}\sum_{x_{1}}\sum_{x_{2}}c\left(x_{1}\right)\beta V\left(x_{1}-x_{2}\right)c\left(x_{2}\right)+\sum_{x}\left[\beta f_{h}\left(c\left(x\right),\rho \left(x\right)\right)-\beta \mu \rho \left(x\right)\right]$$

where

(9) $$\beta f_{h}\left(c,\rho \right)=\frac{\rho +c}{2}\ln\left(\frac{\rho +c}{2}\right)+\frac{\rho -c}{2}\ln\left(\frac{\rho -c}{2}\right)+\left(1-\rho \right)\ln\left(1-\rho \right)$$

is the free energy density of the non-interacting lattice gas mixture in $k_{B}T$ units, and represents the entropic contribution to the grand potential functional.

In the case of the disordered phase with mobile aggregates, the ensemble-averaged concentration is $\langle \hat{c}\left(x\right)\rangle =0$ when $\mu_{1}=\mu_{2}=\mu$. For large densities, however, the probability distribution for c(x) may have two symmetric maxima in each lattice cell, one at c>0 and the other at c<0, corresponding to a cell occupied by the first or the second component, respectively. When the most probable value of c(x) differs significantly from the average value (here, zero), its variance should be taken into account. To include contributions to the grand potential functional from the fluctuations ϕ(x) of the local concentration c(x), we consider the following functional,

(10) $$\beta \Omega \left[c,\rho \right]=\beta \Omega^{MF}\left[c,\rho \right]+\beta \Delta \Omega_{fluc}\left[c,\rho \right],$$

and approximate the fluctuation contribution according to the Brazovskii field theory [41], further developed and extended to a binary symmetrical mixture in Refs. [28,40]. In this approximation,

(11) $$\beta \Delta \Omega_{fluc}\left[c,\rho \right]\approx -\ln\left[\int D\phi \exp\left(-\beta H_{f}\right)\right]$$

where ∫Dϕ denotes a functional integral, and

(12) $$\begin{array}{c} \beta H_{f}\approx \frac{1}{2}\int_{-\pi }^{\pi }\frac{dk_{1}}{2\pi }\int_{-\pi }^{\pi }\frac{dk_{2}}{2\pi }\tilde{\phi }\left(k\right)\beta \tilde{V}\left(k\right)\tilde{\phi }\left(-k\right)\\ +\sum_{x}\left[\frac{\phi^{2}}{4}\left(\frac{1}{\rho +c}+\frac{1}{\rho -c}\right)+\frac{\phi^{4}}{4!}\left(\frac{1}{{\left(\rho +c\right)}^{3}}+\frac{1}{{\left(\rho -c\right)}^{3}}\right)\right]. \end{array}$$

In this model, fluctuations of the local density ρ are not energetically favored (see (8)), and are neglected in the approximate theory.

We are interested in the structure described by the correlation function for the concentration fluctuations,

(13) $$G\left(x_{1},x_{2}\right)=\langle c\left(x_{1}\right)c\left(x_{2}\right)\rangle .$$

In the Fourier representation and self-consistent Gaussian approximation developed in Ref. [40], we have the following:

(14) $$\tilde{G}{\left(k\right)}^{-1}=\frac{\delta^{2}\left(\beta \Omega \right)}{\delta \tilde{c}\left(k\right)\delta \tilde{c}\left(-k\right)}\approx \beta \tilde{V}\left(k\right)+\frac{1}{{\bar{\rho }}_{R}},$$

where $\tilde{c}\left(k\right)$ and $\tilde{G}\left(k\right)$ are the concentration and the concentration–concentration correlation functions in the Fourier representation, respectively, and

(15) $$\frac{1}{{\bar{\rho }}_{R}}=\frac{1}{\bar{\rho }}+\frac{{\langle \phi^{2}\rangle }_{G}}{{\bar{\rho }}^{3}}.$$

In the above, $\bar{\rho }$ is the average density that, for a given μ, satisfies the following equation:

(16) $$\frac{\delta (\beta \Omega )}{\delta \rho }\approx \frac{\delta (\beta \Omega^{MF})}{\delta \rho }+{\langle \frac{\delta \beta H_{G}}{\delta \rho }\rangle }_{G}=0,$$

and the Gaussian approximation,

(17) $$\begin{array}{c} \beta H_{f}\approx \beta H_{G}=\frac{1}{2}\int_{-\pi }^{\pi }\frac{dk_{1}}{2\pi }\int_{-\pi }^{\pi }\frac{dk_{2}}{2\pi }\tilde{\phi }\left(k\right)\tilde{G}{\left(k\right)}^{-1}\tilde{\phi }\left(-k\right) \end{array}$$

has been used. ${\langle X\rangle }_{G}$ denotes the average of the quantity *X* with the probability proportional to $\exp(-\beta H_{G})$. The last terms in (15) and (16) follow from the fluctuation contribution to Ω in (10), and in the MF approximation they are neglected.

The local concentration variance, $\langle \phi^{2}\rangle$, increases when the segregation of the components in the regions of size $∼\pi /k_{b}$ becomes stronger. In the approximation developed in Ref. [40] and adopted here to the 2D lattice, the self-consistent equation for ${\langle \phi^{2}\rangle }_{G}$ is as follows:

(18) $${\langle \phi^{2}\rangle }_{G}=\int_{-\pi }^{\pi }\frac{dk_{1}}{2\pi }\int_{-\pi }^{\pi }\frac{dk_{2}}{2\pi }{\left[\beta \tilde{V}\left(k\right)+\frac{1}{\bar{\rho }}+\frac{{\langle \phi^{2}\rangle }_{G}}{{\bar{\rho }}^{3}}\right]}^{-1}.$$

The stability range of the disordered phase is determined by the sign of the second functional derivative of Ω, which is positive or negative when the disordered phase is stable or unstable, respectively. At the continuous transition to a phase with the oscillating ensemble-averaged concentration, $\delta^{2}\left(\beta \Omega \right)/\left(\delta \tilde{c}\left(k_{b}\right)\delta \tilde{c}\left(-k_{b}\right)\right)=0$. In MF, the corresponding λ-line is just given by the following formula:

(19) $$\frac{\delta^{2}\left(\beta \Omega^{MF}\right)}{\delta \tilde{c}\left(k_{b}\right)\delta \tilde{c}\left(-k_{b}\right)}=0.$$

When fluctuations are taken into account within the self-consistent Gaussian approximation, from (14), (15), and (18), we obtain the following equation for the continuous transition:

(20) $$|\beta \tilde{V}\left(k_{b}\right)|-{\bar{\rho }}^{-1}=\frac{k_{B}T}{{\bar{\rho }}^{3}}\int \frac{dk}{{\left(2\pi \right)}^{d}}{\left[\tilde{V}\left(k\right)-\tilde{V}\left(k_{b}\right)\right]}^{-1}.$$

Importantly, the above equation has or does not have solutions when the integral on the right-hand side is finite or diverges, respectively. It depends on the shape of $\tilde{V}\left(k\right)$ and the dimension, *d*, of the system. In 3D, the integral in Equation (20) is infinite for the Brazovskii functional of an arbitrary order parameter [41], in particular, when applied to block-copolymers [42]. The RHS in (20) diverges for the SALR (mermaid) model [39], the 3D ‘two mermaids and a peacock’ model [28], and for oppositely charged hard spheres of the same size (restricted primitive model, RPM) in 3D continuum space [43]. In all these cases, the fluctuation-induced first-order phase transition takes place instead of the continuous transition, in agreement with simulations and experiments. For the RPM on a simple cubic lattice, however, the integral in (20) is finite [43,44], and a continuous transition between disordered and ordered phases takes place at a temperature significantly lower for a given ρ than predicted in MF.This prediction was confirmed by simulations as well. In this work, we calculate the correlation function, the MF λ-line, and discuss the effect of fluctuations on the stability of the disordered phase and the average density for a given μ.

### 2.3. Simulation Methods

It is recognized that the role of density fluctuations in particle-based models defined on a lattice can be described by means of Monte Carlo simulations. In an attempt to confirm some of the theoretical predictions described above, we performed grand canonical MC (GCMC) simulations of the model defined through Equations (1) and (2) on a triangular lattice, for the particular choice of $J\equiv J_{2}/J_{1}=3.0$. As in our previous work [38], we define at every lattice site an Ising-like variable, *s*, assuming the values +1,−1,0 to represent species 1 and 2, and an empty site, respectively. The dynamic evolution of the model is implemented through the use of particle insertion and removal moves for each species, based on a standard Metropolis scheme.

Following the strategy successfully applied to the one-component system [7], the parallel tempering (or replica exchange) sampling technique was adopted, either at fixed temperature or at fixed chemical potential variables. Within this scheme, a simulation run at a given constant chemical potential, μ, for example, will involve taking $n_{b}$ successive values of the inverse temperature, β, as $\beta_{k}=k\;\delta \beta$ and *k* = 0, 1, 2, …, $n_{b}-1$, with δβ typically in the range of δβ=0.01−0.1. Once a certain number of cycles for the $n_{b}$ cases are completed, we attempt to sequentially interchange configurations between pairs of neighbor states (2k,2k+1) and (2k+1,2k+2). These *swap* moves are accepted with a probability given by exp(−βΔH), where *H* is the grand canonical energy of the system.

Both thermodynamic and structural properties of the model mixture were studied by applying this methodology. We employed two types of initial configurations for the replicas: for the high-temperature states, the lattice was occupied at random with a probability of 1/3 for each species, while for low temperatures, the configurations corresponding to the stable phases at the ground state were used.

The simulation lengths required to achieve reliable estimates of equilibrium quantities for each system size ranged typically from $10^{7}$ cycles for equilibration plus $10^{7}$ cycles for sampling. In general, more cycles per run are required as the system size, *L*, increases. These rather long simulations were required to ensure good sampling of the properties at low temperatures. Most of the simulations were performed on lattices of lateral size *L* = 80 with periodic boundary conditions. Other values of *L*, from *L* = 40 to *L* = 120, were considered as well for some representative cases in order to consider finite-size effects.

We now introduce the standard thermodynamic functions, computed at finite temperatures, which were employed in the analysis of the simulation output. First, the specific heat $C_{v}\left(T,\mu \right)$ is calculated from the fluctuations of the internal energy, as

(21) $$C_{v}=\frac{1}{L^{2}}\left(\langle E^{2}\rangle -{\langle E\rangle }^{2}\right)$$

where E[{s(x)}] is the energy in the configuration {s(x)}, with {s(x)} being the value of the variable *s* in all lattice sites. The brackets denote ensemble averages and the interaction potential is defined in Equation (2). In a similar fashion, one can define the compressibility κ(T,μ) of the mixture from the fluctuations in the number densities of the two components, as follows:

(22) $$\kappa =N_{1}\;\Delta N_{1}+N_{2}\;\Delta N_{2}$$

with $\Delta N_{i}$ being the variance of the i−type particle number density $N_{i}\equiv L^{-2}\;\sum_{x}{\bar{\rho }}_{i}\left(x\right)$. Since we consider only the symmetric case $\mu_{1}$ = $\mu_{2}$, there will be the constraint $\langle N_{1}\rangle$ = $\langle N_{2}\rangle$ = ρ/2.

The correlations in the particle concentrations can be described by means of the structure factor defined on the lattice:

(23) $$S\left(k\right)=\frac{1}{L^{2}}\langle \sum_{x,y}e^{-ik\cdot (x-y)}\rangle$$

which in turn is directly related to the correlation function, $\tilde{G}\left(k\right)$, defined in Equation (14). The location and the height of the peaks displayed by S(k) at different state points (T,μ) will provide us with useful quantitative information to characterize the degree of order of the resulting aggregation patterns and the nature of the various phases that are expected to appear.

## 3. Results

### 3.1. Theoretical Results

#### 3.1.1. Mean-Field Approximation

We first focus on the MF approximation, and determine the MF boundary of stability of the disordered phase. It is given by ${\tilde{G}}^{-1}\left(k_{b}\right)=0$, but with the local concentration variance neglected in (14). The MF boundary of stability of the disordered phase associated with microsegregation ($k_{b}\neq 0$) is called the λ-line, to distinguish it from the spinodal line associated with macrosegregation ( $k_{b}=0$). In the considered mixture, the λ-line takes the following form (see (14), (4), and (6)):

(24) $${\bar{T}}_{\lambda }\left(\bar{\rho }\right)=V_{b}\bar{\rho },$$

where

(25) $$V_{b}=-2\left[J\cos\left(2k_{b}\right)+\left(2J-1\right)\cos k_{b}-2\cos\left(\frac{k_{b}}{2}\right)\right].$$

For J=3, the decrease in energy when a concentration wave with the wavenumber ($k_{b}$) and amplitude (A=1) is excited is $V_{b}\approx 10.311$ and $k_{b}\approx 1.922$. The λ-line in this case is ${\bar{T}}_{\lambda }\left(\bar{\rho }\right)\approx 10.3\bar{\rho }$.

Note that in the mixture, ${\bar{T}}_{\lambda }\left(\bar{\rho }\right)$ is a linear function of $\bar{\rho }$, whereas in the one-component case, ${\bar{T}}_{\lambda }\left(\bar{\rho }\right)$ has a bell shape [5,6,39]. This difference follows from the instability with respect to concentration fluctuations or density fluctuations in the mixture or the one-component system, respectively, and the role of the entropy of mixing in the former case. A similar result was obtained in MF for a symmetrical mixture with the interactions having the property (1) in 3D continuum space [28,35].

For the interactions defined in (1), the internal energy is independent of ρ. From the necessary condition for the equilibrium in MF, $\partial \beta \Omega^{MF}/\partial \bar{\rho }=0$, we obtain the same result as in the non-interacting one-component lattice gas, as follows:

(26) $$\bar{\rho }=\frac{2e^{\bar{\beta }\bar{\mu }}}{1+2e^{\bar{\beta }\bar{\mu }}}.$$

In the symmetrical case, the concentration in the disordered phase is $\bar{c}=0$.

The λ-line in the $\bar{\mu },\bar{T}$ variables can be obtained from (24) and (26), and has the following form:

(27) $${\bar{\mu }}_{\lambda }\left(\bar{T}\right)=-\bar{T}\ln\left[2\left(\bar{\beta }V_{b}-1\right)\right].$$

**Figure 2:**
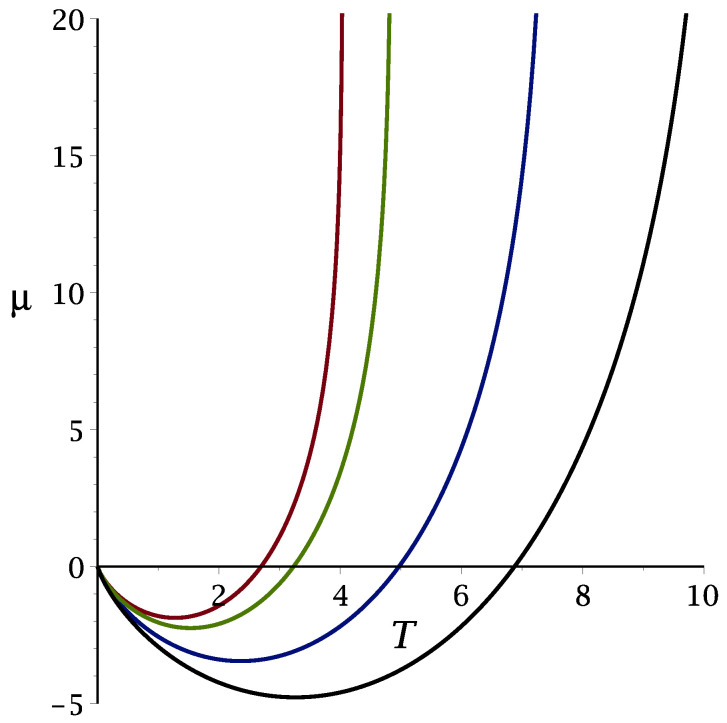
The MF λ-line ${\bar{\mu }}_{\lambda }\left(\bar{T}\right)$ separating the unstable and stable disordered phases for $\bar{\mu }>{\bar{\mu }}_{\lambda }\left(\bar{T}\right)$ and $\bar{\mu }<{\bar{\mu }}_{\lambda }\left(\bar{T}\right)$, respectively. Black, blue, green and red lines (from bottom to top) correspond to J=3,2,1,1/2, respectively.

**Figure 3:**
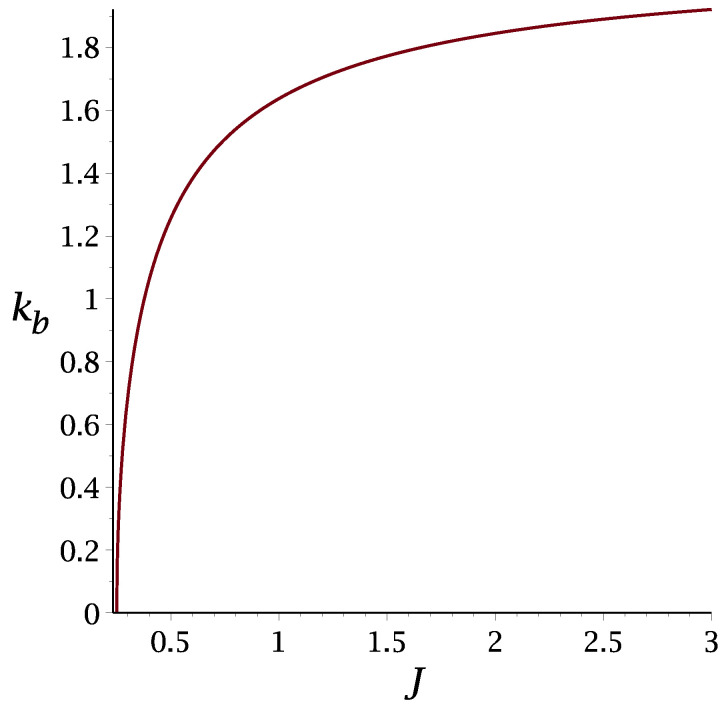
The wavenumber $k_{b}$ (Equation (6)) of the concentration wave leading to the largest decrease in energy, as a function of the ratio $J=J_{2}/J_{1}$ of the interaction between particles occupying the cells separated by Δx = 2 and Δx = 1, respectively.

The λ-line (27) is shown in Figure 2 for J=0.5,1,2,3, and the wavenumber, $k_{b}$, of the energetically favored concentration wave is shown in Figure 3 for 1/4≤J≤3.

#### 3.1.2. Self-Consistent Gaussian Approximation

**Figure 4:**
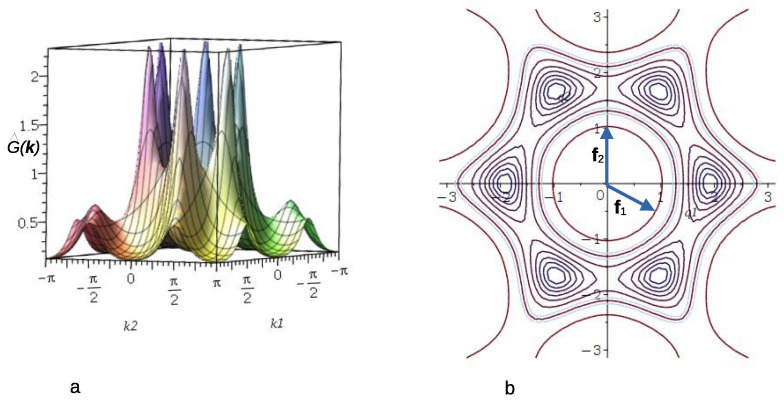
The concentration–concentration correlation function $\tilde{G}\left(k\right)$ (see (14)) for J=3, $\bar{T}=4$ and ${\bar{\rho }}_{R}=1/3$, corresponding to $\bar{\rho }\approx 0.72$. Panel (**a**) shows $\tilde{G}\left(k\right)$ as a function of the coordinates $k_{1},k_{2}$ of $k=k_{1}f_{1}+k_{2}f_{2}$. Panel (**b**) shows a contour plot of $\tilde{G}\left(k\right)$ together with the lattice vectors of the reciprocal lattice. The lattice vectors of the reciprocal, $f_{i}$, and triangular, $e_{i}$, lattices satisfy $f_{i}\cdot e_{j}=\delta_{ij}^{Kr}$, where $\delta_{ij}^{Kr}$ is the Kronecker delta.

Let us now take into account the fluctuations of the local concentration. In order to calculate the correlation function, $\tilde{G}\left(k\right)$, with the local concentration variance included in (14), we numerically solve Equation (18). The obtained correlation function $\tilde{G}\left(k\right)$ in the disordered phase is shown in the Fourier representation in Figure 4 for $\bar{T}=4$ and ${\bar{\rho }}_{R}=1/3$, corresponding to the average density $\bar{\rho }\approx 0.72$. Note that the point $\left(\bar{\rho },\bar{T}\right)=\left(0.72,4\right)$ lies on the low-$\bar{T}$ high-ρ side of the λ-line obtained in MF (see (24)), signaling that the disordered phase continues to be stable on this side of the MF λ-line.

**Figure 5:**
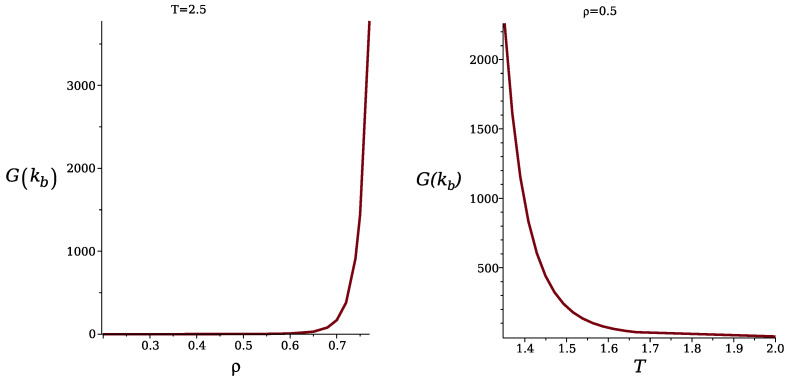
The maximum of the concentration–concentration correlation function in the disordered phase in the Fourier representation, $\tilde{G}\left(k_{b}\right)$ (see (14)), for J=3. $\tilde{G}\left(k_{b}\right)$ is shown as a function of density for $\bar{T}=2.5$ (**left** panel), and as a function of temperature for ρ=0.5 (**right** panel). The dimensionless temperature is $\bar{T}=k_{B}T/J_{1}$.

**Figure 6:**
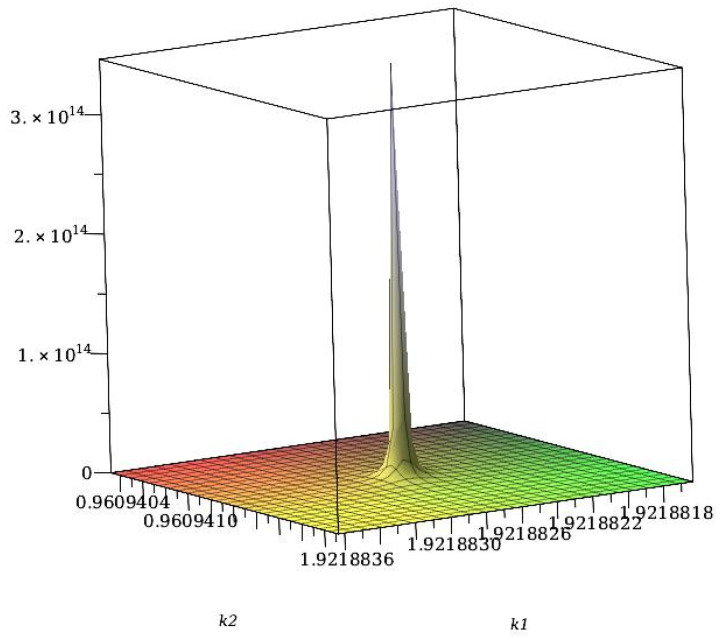
The function $F\left(k\right)={\left[\tilde{V}\left(k\right)-\tilde{V}\left(k_{b}\right)\right]}^{-1}$ for $k\approx k_{b}$. The region around each peak of F(k) provides a divergent contribution to the integral ∫dkF(k), and according to the self-consistent Gaussian approximation, there is no instability of the disordered phase with respect to the oscillatory concentration for the finite *T*, as Equation (20) has no solutions for T>0.

In Figure 5, we show the maximum of the correlation function, $\tilde{G}\left(k_{b}\right)$, as a function of density for the fixed temperature $\bar{T}=2.5$, and as a function of $\bar{T}$ for fixed ρ=0.5. Note the rapid increase in $\tilde{G}\left(k_{b}\right)$ with increasing density or decreasing temperature, reaching very large, yet finite values. In fact, $\tilde{G}\left(k_{b}\right)$ diverges only for T=0, indicating that within the considered Gaussian approximation, the disordered phase is stable, although the second functional derivative of the grand potential, $1/\tilde{G}\left(k_{b}\right)$, is very small for the shown range of parameters, and decreases to values much lower than $10^{-3}$ for larger ρ and/or smaller *T*. To confirm that $\tilde{G}{\left(k_{b}\right)}^{-1}>0$ and no continuous transition to a stripe phase is obtained in this approximation, we focus on Equation (20), and estimate the integral of $F\left(k\right)={\left[\tilde{V}\left(k\right)-\tilde{V}\left(k_{b}\right)\right]}^{-1}$. The function F(k) for $k\approx k_{b}$ is shown in Figure 6. F(k) diverges for $k=k_{b}$, and upon a suitable change of variables, we find that the contribution to the integral of F(k) from the neighborhood of each $k=k_{b}$ is proportional to $\int_{0}^{\delta }qdq/q^{2}=\infty$. Note that the dimensionality of the system plays an important role for ∫dkF(k).

We should note that the lack of instability of the disordered phase means that this phase is either stable or metastable, and the transition to a phase with oscillations of the ensemble-averaged concentration is of the first order.

In order to interpret the MF λ-line within the present Gaussian approximation, we note that $\exp[-\frac{1}{2}|\tilde{\phi }\left(k_{b}\right){|}^{2}\left(\beta \tilde{V}\left(k_{b}\right)+1/\rho \right)]$ (see (12)) is the ratio between the probability of the oscillatory concentration with the optimal wavelength and the amplitude $\tilde{\phi }\left(k_{b}\right)\ll 1$, and the probability of an isotropic distribution of the particles in instantaneous states. This ratio of probabilities is larger than one for $\beta \tilde{V}\left(k_{b}\right)+1/\rho <0$. Thus, the λ-line obtained in MF marks the structural change in typical snapshots from isotropic structures to alternating stripes, but the orientation and location of the stripes and the defects in different regions and different snapshots are different. After ensemble averaging, an isotropic structure emerges. Importantly, however, the MF λ-line provides the first estimation of the phase-space region where the ordered phases may appear, serving as a useful guide for simulations.

**Figure 7:**
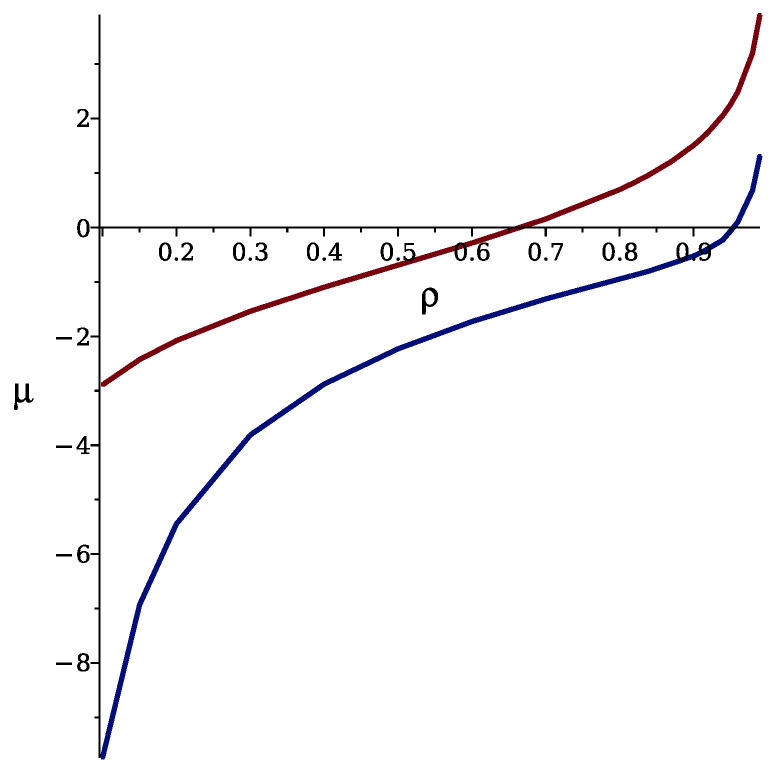
The chemical potential, μ, in $k_{B}T$ units for J=3 and $\bar{T}=4$ in the disordered phase (stable or metastable) as a function of density. The upper red and the lower blue curves correspond to the fluctuation contributions to the grand potential neglected and included, respectively.

In Figure 7, we compare the chemical potential as a function of density with the fluctuation contribution to the grand potential neglected and included. With the fluctuation contribution neglected, i.e., in MF, the chemical potential depends on density in the same way as in the non-interacting mixture, due to the symmetry of interactions. With the fluctuation contribution included, we obtain from Equation (16) the following equation for the stable or metastable disordered phase:

(28) $$\beta \mu \approx \ln\left(\frac{\rho }{2(1-\rho )}\right)-\frac{{\langle \phi^{2}\rangle }_{G}}{2\rho^{2}}-\frac{3{\langle \phi^{2}\rangle }_{G}^{2}}{4\rho^{4}}.$$

We can see that with the concentration fluctuations taken into account, the density for a given μ is significantly larger than in MF, or a given ρ is obtained for a smaller μ than expected from MF. This is because MF is based on the average concentration that vanishes, and the stripe formations in instantaneous states of the disordered phase are disregarded. When the local concentration variance associated with the formation of alternating stripes in mesoscopic subsystems is taken into account, the sum of attractive interactions overcomes the repulsive ones, and more particles can be accommodated in the system for a given μ. Thus, concentration fluctuations in this model lead to the same effect as attractive interactions in one-component systems.

Let us finally comment that our results were obtained in an approximate theory. Since $\tilde{G}{\left(k_{b}\right)}^{-1}$ assumes very small values for large ρ and small $\bar{T}$, we cannot entirely exclude the possibility that the exact result for the fluctuation correction in Equation (15) is smaller than ${\langle \phi^{2}\rangle }_{G}/{\bar{\rho }}^{3}$ predicted in the self-consistent Gaussian approximation, and $\tilde{G}\left(k_{b}\right)$ diverges for $\bar{T}>0$. Moreover, we did not take into account the orientational ordering of mobile elongated clusters (stripes), i.e., broken rotational symmetry but not translational symmetry.

### 3.2. Simulation Results

In this section, we compile the outcome of grand canonical Monte Carlo simulations performed at several representative points in the T−μ plane. Most of the simulations employ triangular lattices of lateral dimension *L* = 80, and in certain cases, other sizes were also considered for testing finite-size effects.

First, we briefly summarize the findings of our previous work on the binary system [38], focused on the properties of the ground state. In contrast to the one-component system [7], the addition of the second component leads to the formation of small clusters that aggregate and create more dense and ordered domains, even at rather low chemical potentials.

**Figure 8:**
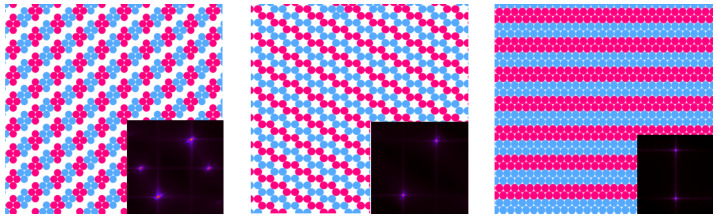
Schematic cartoons of the lattice configurations in the calculated ground states (GSs) of the binary mixture with J=3.0 and $\mu_{1}=\mu_{2}=\mu$. From left to right: the (CC) phase, (ZZ) phase, and (*L*) phase. Red and blue circles represent particles of the first and the second component, respectively, and white regions represent cells occupied by the solvent. For more details, see Ref. [38]. At the bottom right corner of each panel, the structure factor S(k) for the corresponding simulated state is included.

Based on the GS analysis of the mixture for the symmetric case, $\mu_{1}=\mu_{2}=\mu$, and with a strong repulsion, J=3.0, we expect the presence of several periodically ordered phases at sufficiently low temperatures. Lamellas (denoted by L) are stable for $\bar{\mu }\geq -2.0$, while two different ordered phases (denoted CC and ZZ) minimize the thermodynamic Hamiltonian, *H* (Equation (3)) in the range of $-5.0\leq \bar{\mu }\leq -2.0$. The former is composed of alternating adjacent bilayers of the first and second components, with ρ≃1, while the CC (“cluster chain”) is made of chains of alternating clusters of the two types separated by empty layers. The ZZ (“zig-zag cluster”) phase, on the other hand, consists of alternating zig-zag chains of the first and second components. We note that, as the predicted ground states are degenerated, CC and ZZ phases can coexist for the entire stability range. These periodic, ordered phases predicted for T=0 are schematically shown in Figure 8, together with the corresponding structure factor for each case. The presence and location of the peaks in S(k) in these patterns can be rationalized by taking into account the unit cell shape and size in the real space for the different periodic structures.

It is generally expected that the degeneracy of the ground state will influence the phase behavior for T>0, an effect that cannot be properly described by the approximate theory. Thus, Monte Carlo simulations can, in this case, provide useful insight into the thermodynamics and structures at finite temperatures.

#### 3.2.1. Structure at High Temperatures: The Disordered Fluid

**Figure 9:**
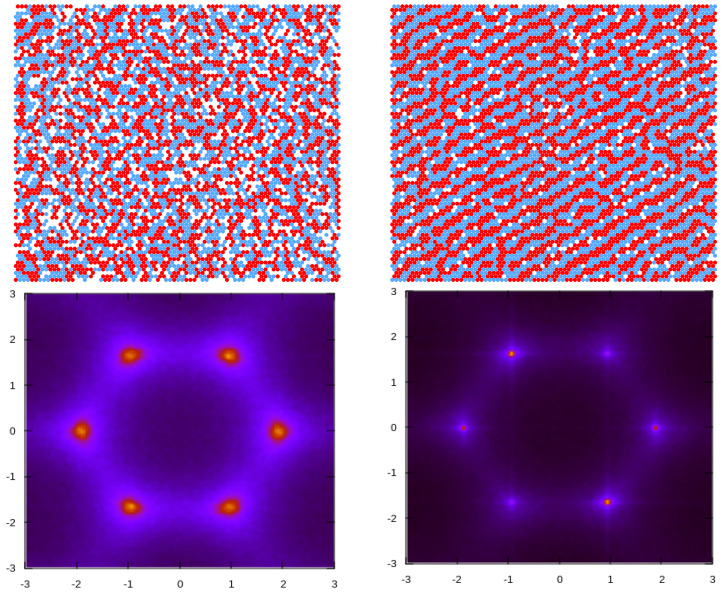
Structure of the mixture from the MC simulation at a fixed chemical potential, μ = 4.0. Top row: representative configurations are shown for $\bar{T}=3.08$ (**left**) and $\bar{T}=2.86$ (**right**), along with their respective concentration structure factors, S(**k**) (**bottom** row). Red and blue circles represent particles of the first and the second component, respectively, and white regions represent cells occupied by the solvent. The dimensionless temperature is $\bar{T}=k_{B}T/J_{1}$, where $J_{1}$ is the nearest-neighbor attraction.

In the disordered phase, at high temperatures and high chemical potential, the average density becomes spatially homogeneous, and the formation of small clusters made of both species, with different sizes and shapes, is energetically favored; this is due to the range of the repulsive (attractive) part of the interaction potential for like (unlike) particles. This equilibrium cluster phase (see Figure 9, top left panel), which we identify as the fluid (F) phase, will eventually undergo a transition to an ordered phase as the temperature decreases.

The isotropic character of the disordered fluid phase is reflected in the pattern displayed by the concentration–concentration structure factor, S(*k*) (Figure 9, bottom left panel), where rather broad peaks with six-fold symmetry are present around finite wave vectors with wavenumber $k_{b}$, which is much smaller than that corresponding to nearest-neighbor distance. It is known from previous works on continuum systems that the appearance of this characteristic peak is a signature of the development of intermediate-range order in the mixture [15]. Notably, a closer inspection of the scattering patterns S(**k**) yields a value of $k_{b}\approx 1.92$, which is very close to the value of 1.922, as predicted by the theory in Section 3.1.2 (see Figure 4b and Figure 6).

Upon slightly decreasing temperatures, the disordered fluid undergoes a structural change. Now, the structure contains alternating stripes of the two components with different sizes but similar thicknesses. Despite the lack of periodicity in all three lattice directions, one direction (see Figure 9 top right panel) can still be distinguished in the individual configurations. This non-isotropic phase, characterized by alternating stripes of the first and second components of different lengths, is termed a *stripe fluid* (SF).

The observed sudden change in the concentration pattern after a mild variation in temperature is reflected in the structure factor shown in Figure 9 (bottom right panel), which displays six-fold peaks at $k_{b}$, much sharper than before. However, the origin of these peaks is the result of an ensemble average taken from a collection of microstates that individually present a preferential direction for the stripes, as can be seen in the example snapshot considered.

**Figure 10:**
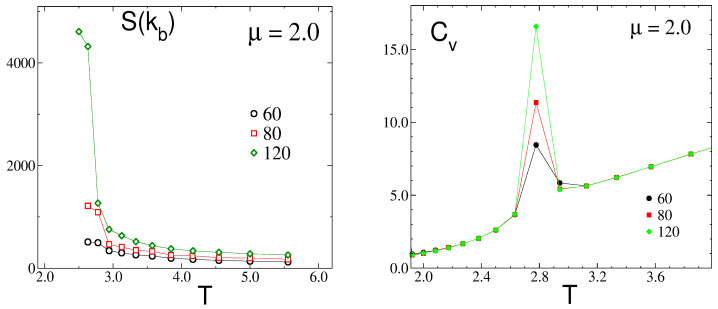
(**Left**) the evolution of S($k_{b}$) with the temperature, in the disordered fluid phase (F) and at a fixed chemical potential, μ = 2.0. (**Right**) the dependence of the specific heat, $C_{v}$, on the temperature. Several lattice (L) sizes are shown, as indicated. The dimensionless temperature is $\bar{T}=k_{B}T/J_{1}$ and the dimensionless chemical potential is $\bar{\mu }=\mu /J_{1}$, where $J_{1}$ is the nearest-neighbor attraction. $S(k_{b})$ is in units of $1/a^{2}$, where *a* is the lattice constant, and $C_{v}$ is in units of $k_{B}T$.

The evolution of the maximum S($k_{b}$) of the structure factor with the temperature at a fixed chemical potential $\bar{\mu }$ = 2.0 is shown in Figure 10 (left panel). Starting from high temperatures in the fluid phase (F), the peak at $k_{b}$ becomes sharper and higher as the temperature decreases until it reaches a maximum (but still finite) value at a certain temperature, denoted as ${\bar{T}}_{x}\approx 2.75$. Below that temperature, the anisotropic stripe fluid (SF) structure begins to develop.

This behavior, together with a detailed inspection of representative configurations along the temperature path, led us to identify this transition point as a sharp crossover between isotropic and anisotropic structures in the fluid phase. Additional evidence for this hypothesis is provided by the pronounced maxima observed in the specific heat, $C_{v}$, for $\bar{T}\approx {\bar{T}}_{x}$; see the right panel in Figure 10. This fact denotes strong fluctuations in the internal energy of the mixture that can be related to the observed rearrangements of defects involved in the structural transition. Furthermore, the increase in the height of the peaks for larger lattice sizes points toward a continuous phase transition. However, the available simulation data, based on rather modest lattice sizes, prevent us from asserting it. At this stage, the question if the sharp structural crossover is associated with a continuous phase transition between isotropic and anisotropic phases (in a thermodynamic sense) remains open.

**Figure 11:**
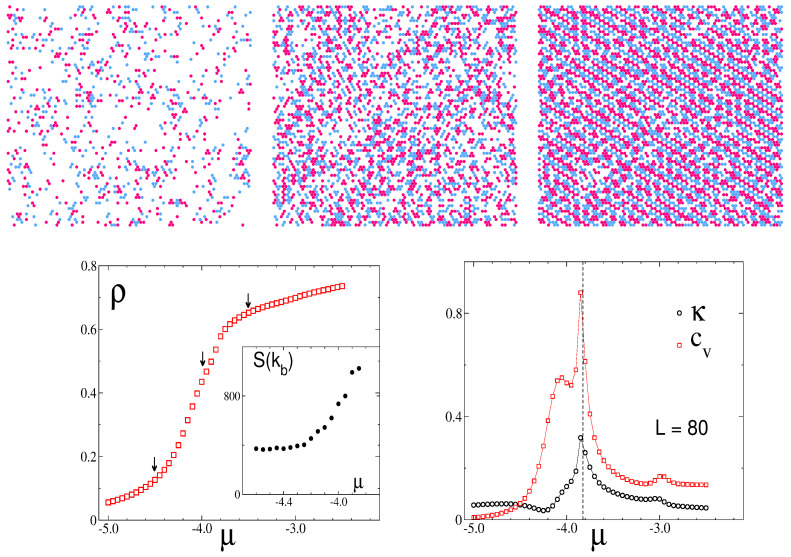
Monte Carlo results in the low-μ region, at a fixed temperature, $\bar{T}$ = 1.4. (**Top**) snapshots for $\bar{\mu }=-4.5,-4.0,-3.5$, from left to right respectively. Red and blue circles represent particles of the first and the second component, respectively, and white regions represent cells occupied by the solvent. (**Bottom**) isotherms $\rho (\bar{\mu })$ (**left** panel) and the compressibility, κ, and specific heat, $C_{v}$ (**right** panel, with both quantities rescaled). Inset: the value S($k_{b}$) as a function of $\bar{\mu }$. In the **left** panel, arrows indicate the state points that correspond to the snapshots shown above. In the **right** panel, the dashed line indicates the location of $T_{x}$ (for more details, see the text).

Now, we focus on behavior at a low chemical potential and explore the effects it has on the stability of the disordered fluid, at a fixed temperature of $\bar{T}$ = 1.4. By inspecting the snapshots in Figure 11, top row, we can see the stripe pattern on the right for $\bar{\mu }=-3.5$, and the isotropic pattern for $\bar{\mu }=-4.5,-4.0$. Notably, the λ-line that—according to our theory beyond MF—indicates the change from the most probable isotropic pattern to the most probable stripe pattern in a single snapshot, Equation (27), is $\bar{\mu }\left(1.4\right)\approx$−3.56. Thus, the theoretical prediction concerning the instantaneous patterns agrees with the simulation results.

The increase in density with chemical potential, as shown in Figure 11 (bottom left panel), coincides with the development of the structure in the mixture. A comparison of these isotherms with the corresponding isotherms calculated theoretically, like those included in Figure 7, indicates the same trend in the curves.

On the other hand, additional evidence for the F-SF transition appears in Figure 11 (bottom right panel), where the specific heat, $C_{v}$, and compressibility, κ, are shown as functions of μ. Both quantities display peaks at μ≈ −3.8, denoting strong fluctuations in the internal energy and density, respectively. The increase in the peak of the structure factor at $k_{b}$ in Figure 11 (inset in the bottom left panel) also denotes the proximity to the transition, but this time, the variation is quite smooth.

**Figure 12:**
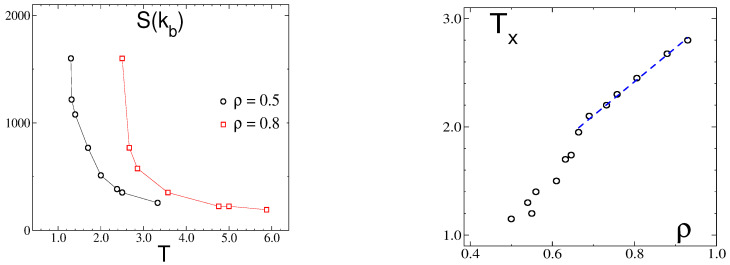
(**Left**) the maximum value S($k_{b}$) of the structure factor as a function of temperature for fixed densities, ρ = 0.5 and 0.8, as indicated. (**Right**) the temperature $T_{x}$ of the structural crossover as a function of the density. The linear fit in the range of ρ≥ 0.7 yields a slope of 3.0.

Finally, we consider the dependence of S($k_{b}$) on temperatures for fixed values of the mixture density, as shown in Figure 12 (left panel). These data points are collected from the different sets of GCMC simulations performed either at fixed *T* or at fixed μ, as the average density is an output in these types of simulations. For the two densities in the plot, ρ = 0.5 and ρ = 0.8, the rapid increase in S($k_{b}$) indicates the approach to the F-SF transition; however, the character of this transition cannot be determined from these types of data. In any case, a rough estimate for the temperature, $T_{x}$, can still be obtained, and these data points are collected in Figure 12 (right panel), where $T_{x}$ is plotted as a function of ρ. This curve shows a linear dependence for large enough densities, ρ≥ 0.7, in agreement with the theoretical prediction of Equation (24) but with a much smaller slope, around +3.0, than in the MF approximation. The discrepancy between both results is assigned to the mean field theories, which overestimate the transition temperatures.

Overall, the analysis of the simulation data performed above indicates that the nature of this sharp structural crossover remains elusive with the available information at present, and more accurate simulation data, as well as the theory accounting for the orientational ordering of mobile stripes, are clearly needed.

#### 3.2.2. Phase Transitions of Lamellas

In order to characterize the presence and degree of the order of lamellas (in the mixture) at higher chemical potentials, we performed parallel tempering simulations at fixed $\bar{\mu }\geq$ 0.0, applying different configurations as the initial conditions. Thus, one set of simulations began at a very high temperature, $\bar{T}$ = 50.0, and finished at a low temperature, typically *T* = 0.2. As the initial configurations for all the replicas, we used those corresponding to the limit of infinite temperature and finite $\bar{\mu }$ (i.e., a fully disordered state). On the other hand, for the ordered lamellar phase at low temperatures, we started simulations at $\bar{T}$ = 0.1, with initial configurations taken as perfectly oriented lamellas corresponding to the *T* = 0 ground state.

**Figure 13:**
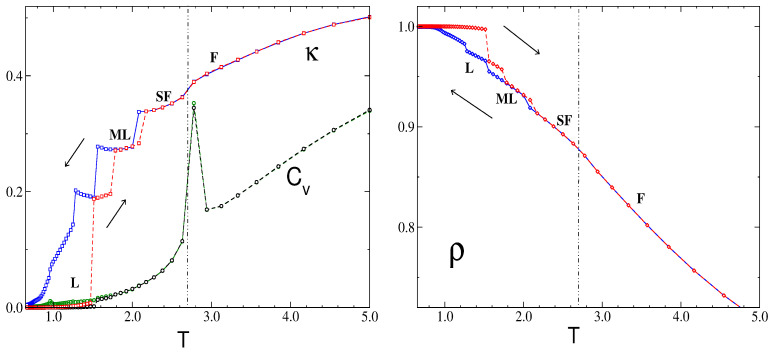
Progression of transitions observed from the lamellar region up to the disordered fluid. (**Left**) the dependence of the specific heat ($C_{v}$) and compressibility (κ) on temperature, for a sample of size *L* = 80 at a fixed chemical potential, μ = 2.0. Both magnitudes are rescaled for clarity. (**Right**) an analysis of the density (ρ). In both plots, different types of lines indicate the corresponding initial conditions applied in the simulations: disordered fluid (full lines)/ordered lamellas (dashed lines). The dot-dashed vertical lines indicate the $T_{x}$, i.e., the maximum of the structure factor, $S(k_{b})$, as a function of $\bar{T}$ for fixed $\bar{\mu }$.

In Figure 13, we show results for the cases of $\bar{\mu }$ = 2.0 and L = 80. Upon raising the temperature from its lowest value, at some temperature, the ordered lamellas melt irreversibly to produce phases that gradually loose their translational and rotational order. We found that the melting temperature for the ordered lamellas is relatively low, $\bar{T}\approx$ 1.5 for $\bar{\mu }$ = 2.0, and this melting transition is first-order, as seen in the large hysteresis in the density and the finite jump in the compressibility. As seen in Figure 13, there are two first-order transitions at low $\bar{T}$, L-ML1 and ML1-ML. In both ML1 and ML, stripes in the direction perpendicular to one of the lattice vectors are formed, but the compressibility in the less ordered ML phase is larger.

With an increase in temperature, the molten lamella slowly begins to disaggregate, while the orientational order decreases until another jump in κ occurs, and a stripe-fluid phase with shorter stripes and a larger number of defects develops. There is also a small variation in ρ at the transition; however, the energy and its fluctuations behave smoothly. Likewise, there is no significant difference between $S(k_{b})$ in SF and ML phases.

Finally, an additional increment in *T* induces a sharp structural crossover of the SF into a fully disordered, isotropic phase. At this temperature, the specific heat presents a pronounced maximum, which depends on the system size, L (as seen in Figure 10, where this temperature is already denoted, $T_{x}$). Also, the compressibility shows a maximum at $T_{x}$, although it is less pronounced. On the other hand, the energy per particle and the density both appear to be continuous across $T_{x}$. This fact makes it difficult to accurately identify the nature of the transition between the anisotropic and disordered isotropic fluid.

We note that the temperature of the sequence of transitions (F-SF, SF-ML, ML-L) observed in Figure 13 depends on the chemical potential, and for all of them, it assumes the maximum for $\bar{\mu }\approx$ 10.0.

**Figure 14:**
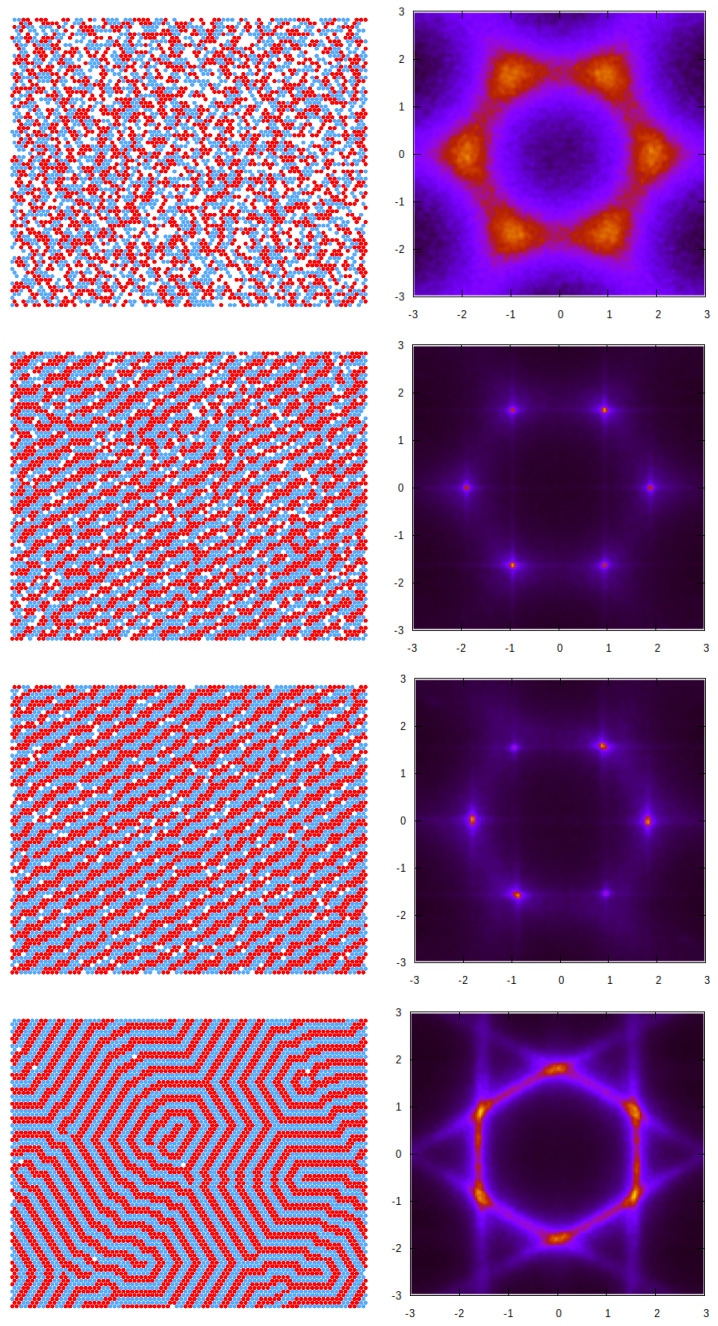
Structural evolution with the temperature at a fixed chemical potential of μ = 2.0, starting from a disordered state at *T* = 50.0. (**Left**) typical configurations at selected temperatures. Red and blue circles represent particles of the first and the second component, respectively, and white regions represent cells occupied by the solvent. (**Right**) corresponding structure factor S(**k**). From top to bottom, the temperatures (densities) are 5.0 (*0.71*), 2.5 (*0.89*), 1.66 (*0.95*), and 0.91 (*0.99*). The phases that can be identified are as follows: fluid (F), stripe fluid (SF), molten lamella (ML), and lamella (L), respectively.

Some of the features described above are observed in the structural evolution of the system upon a quenching started from a very high temperature, as shown in Figure 14, for the case $\bar{\mu }$ = 2.0, where some representative configurations are included together with their corresponding averaged structure factors. In the top panel, a configuration in the isotropic fluid (F) phase at a high temperature is presented. The microscopic state consists of small clusters with a broad distribution of sizes and shapes, at a relatively high density. This is also demonstrated by the broad peaks in S(**k**), which display the six-fold symmetry and are located at k = $k_{b}$.

For a temperature just below $T_{x}$ in the second panel, stripes of different lengths, and other cluster structures with a local period of around 4 in one direction, are observed. The collection of these microstates with broken rotational symmetry (SF-phase) produces an ensemble-averaged S(**k**) that still has six-fold symmetry but with much sharper peaks.

Upon decreasing the temperature further, in the third panel, a typical molten lamella (ML) configuration is presented, where lamellar pieces have preferential orientations and create a percolating structure with a distinguished direction. Like in the previous case, the possible orientations of the stripes are equally probable, and ensemble averaging leads to six peaks. However, we verified that all individual snapshots show an anisotropic structure.

Finally, in the lower panel, the ordered lamella (L) structure is presented, which still contains a few defects that are inherent to thermal fluctuations. However, the structure is quite regular, the translational order is high and the density is close to unity. The different orientations of the well-defined, period-$2\sqrt{3}$ stripes running along the lattice axis produce the pattern displayed by S(**k**) with peaks located at vectors $k_{L}$, which are different from the $k_{b}$ found for the remaining phases.

## 4. Discussion

We studied a triangular lattice model with cells that can be occupied either by a solvent or by a particle of the first or second components. The model is suitable for a substrate with adsorption centers forming a triangular lattice. The interaction potential between like-adsorbed particles, Equation (2), is attractive for nearest neighbors and repulsive for third neighbors, and the cross-interaction is of the opposite sign. For such interactions, the ordered patterns shown in Figure 8 are energetically favored for equal chemical potentials of the two components. We explored the structural evolution from the disordered fluid to ordered patterns by decreasing the temperature at a fixed chemical potential, and by increasing the chemical potential (or density) at a fixed temperature.

In the disordered phase stable at a high temperature or for a strongly negative chemical potential, the ensemble-averaged density and concentration are independent of the position. We determined the structure factor of this phase within the mesoscopic theory at the level of the self-consistent Gaussian approximation and by MC simulations. In the simulations, important information is also provided by inspecting individual snapshots.

The structure factor shows that the underlying lattice leads to a significant difference compared to the continuous space. In continuum, the structure is isotropic, and on the lattice, six orientations are distinguished. This leads to six peaks of the structure factor in our model. The vectors, $k_{b}$, corresponding to the maxima of the structure factor, in the theory (Figure 4 and Figure 6) and simulations (Figure 9), are $k_{b}\approx \pm 1.92{\hat{e}}_{i}$, where ${\hat{e}}_{i}$ denotes the lattice vectors. Thus, the most probable concentration oscillations are in a direction parallel to one of the lattice vectors.

The maxima of the structure factor are rather broad for high $\bar{T}$ or small $\bar{\mu }$, and the corresponding snapshots show the formation of alternating clusters of the two components having similar thicknesses and different lengths (Figure 9 and Figure 11). The stripes are perpendicular to one of the lattice vectors but are much shorter than the size of the system, and the resulting pattern is isotropic (see Figure 9, Figure 11 and Figure 14).

When the density increases at a fixed, sufficiently low temperature, or when the temperature decreases at a fixed, sufficiently high density, the maximum of the structure factor rises to very large values, but we did not observe a divergence that would signal a continuous transition between phases with and without periodic modulations of the concentration (Figure 5 and Figure 10). The maximum of the structure factor obtained in the theory is in semiquantitative agreement with the results of the simulations, as can be seen for ρ=0.5 from Figure 5, right panel, and Figure 12, left panel. Full quantitative agreement is not expected, because on the one hand we made several approximations in the theory, and on the other hand, systems with finite sizes were considered in simulations, with significant dependence of $S(k_{b})$ on the system size (Figure 10).

The very high and narrow peaks of S(k) are associated with alternating long stripes perpendicular to one of the lattice vectors (Figure 5 and Figure 10), and in every single snapshot, the pattern formed by the particles is anisotropic (Figure 9; see Figure 11, top panel, and Figure 14). The stripes are not smooth and their lengths increase with decreasing $\bar{T}$. Each allowed orientation of the stripes can be chosen with equal probability; therefore ensemble averaging leads to a symmetrical structure factor with six peaks.

On the level of the structure factor, the isotropic and anisotropic patterns in individual snapshots are associated with broad maxima of S(k) or with maxima that shrink to very small spots, respectively, as shown in Figure 9, Figure 12 and Figure 14. Importantly, the heat capacity, $C_{v}$, assumes a maximum when the structures of individual snapshots change from isotropic to anisotropic (see Figure 10). We should note that the energy is expected to increase with an increasing number of defects in the ordered pattern for which the energy is low. Thus, upon a change from the isotropic snapshots with a large number of defects to the anisotropic snapshots with a smaller number of defects, the energy fluctuations are large. The maximum of $C_{v}$ indicates the structural change in instantaneous states of the disordered phase from the isotropic fluid (F) to the stripe fluid (SF). Density and compressibility change smoothly at this structural crossover, although the slope of κ is larger near the maximum of $C_{v}$ (Figure 13 for $\bar{\mu }$ = 2.0). Further studies are necessary to clarify the nature of this structural change between isotropic and anisotropic structures.

Let us discuss in more detail the structural evolution for decreasing $\bar{T}$ with fixed $\bar{\mu }$. In the case of relatively large chemical potential, $\bar{\mu }$ = 2.0, Figure 13 shows steps in the $\kappa (\bar{T})$ and $\rho (\bar{T})$ curves for $\bar{T}<2.2$. The large decrease in κ and the small increase in density at $\bar{T}\approx 2$ are associated with a transition from the SF to the molten lamella ML phase, with the same orientation of alternating stripes, but with a more ordered pattern, stripes with lengths comparable to *L*, and a somewhat smaller number of empty sites. There is another first-order transition between the less and more ordered ML phases with a small jump in density for increasing temperatures that we observed for different values of $\bar{\mu }$ as well. The most ordered phase with the highest density is the lamellar L phase for $\bar{\mu }$ = 2, with a different orientation of the stripes than in the phases at higher $\bar{T}$. The observed hysteresis suggests first-order transitions at $\bar{T}$ < 2.2.

**Figure 15:**
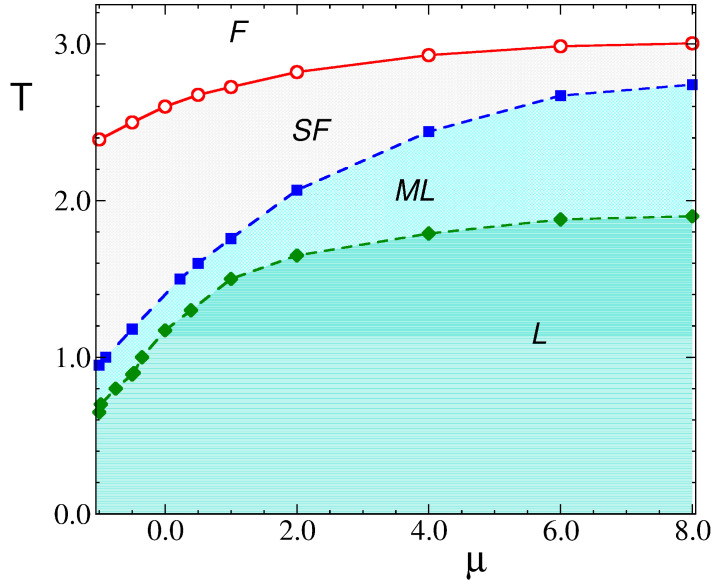
Tentative phase diagram for $J=J_{2}/J_{1}=3.0$. Only the region of positive μ is shown. The full lines (red circles) indicate the loci of $T_{x}$ at structural transitions between the isotropic disordered phase (F) and the stripe fluid (SF). The dashed lines denote first-order transitions: blue squares are for the transition between the stripe fluid (SF) and molten lamellas (MLs), while the green diamonds indicate the melting transition of ordered lamellas (L).

The results discussed above are summarized in a preliminary tentative phase diagram in Figure 15, where we present the outcomes of simulations performed at fixed positive values of the chemical potential. The loci of the several transitions described in previous paragraphs and in Figure 13 are displayed as dashed or full lines. We note that the progression of phases started at low temperatures appears to reach asymptotic values of the transition temperatures for chemical potentials as large as μ≈ 10. On the other hand, the limit of lower (negative) chemical potential where other ordered patterns are expected (i.e., CC and ZZ phases) remains to be explored and will be the topic of future studies.

The structural evolution with increasing chemical potential at a fixed temperature $\bar{T}$ = 1.4 is shown in Figure 11. We see the change in the snapshots from a dilute gas through an isotropic distribution of one-component clusters (F) to alternating stripes that are typically quite long (SF). When the negative chemical potential approaches zero, ρ(μ) isotherms have an S shape (Figure 7 and Figure 11). The density for a given $\bar{\mu }$ is significantly larger than in the non-interacting lattice gas when the self-assembly into clusters or stripes takes place. We obtain a qualitative agreement between the theoretical and simulation results. At the crossover between the isotropic F and anisotropic SF structures, $C_{v}$ as well as κ reach a maximum.

Let us finally comment on the structures expected for different chemical potentials of the two components. In the ground state, the L phase coexists with the hexagonal arrangement of clusters of the minority component in the liquid of the majority component for $\mu_{1}\neq \mu_{2}$. When the difference between the chemical potentials becomes large, only the particles of the component with the larger chemical potential remain and form the same patterns as in the one-component SALR model [38]. The question of how the structural evolution of patterns self-assembled on a pre-patterned surface changes with increasing temperature for $\mu_{1}\neq \mu_{2}$ requires a separate study. We expect some differences between the present case and the continuous model, where longer-range interactions lead to thicker clusters and stripes [30], due to the lattice structure and the range of interactions.

## 5. Conclusions

Our results indicate that in self-assembling systems, the role of mesoscopic fluctuations is crucial for the stability of different phases consisting of a periodic distribution of some structural motifs.

The substrate, pre-patterned into triangular adsorption centers, has a rather strong influence on the self-assembled patterns, particularly on the orientation of the stripes and their smoothness. There are more patterns and transitions between them on the pre-patterned substrate than on the smooth one or in 3D for similar types of interparticle interactions [28,30].

Possible applications of the self-assembled stripes depend on the optical, electromagnetic, heat conductivity, etc., properties of the particles of the two components. From our fundamental studies concerning all types of particles, it follows that for many applications, the optimal structure represents the ML phase (Figure 14). At a rather large chemical potential, all stripes in the ML phase are long and have the same orientation, and a relatively small number of fractures appear. Upon heating, the ML phase undergoes a first-order transition to the SF phase with shorter stripes and a larger number of defects, including branching and dislocations. Thus, it is important to determine the temperature range of the stability of the ML phase in particular systems, since it depends on the strength of the interparticle interactions. The maximum of the specific heat marks the transition between the isotropic and SF phases (Figure 13) and can serve as a helpful indicator that the ML phase is stable at even lower temperatures for a given chemical potential.

## Data Availability

The data supporting the conclusions of this article will be made available by the authors on reasonable request.
